# RANKL Drives Bone Metastasis in Mammary Cancer: Protective Effects of Anti-Resorptive Treatments

**DOI:** 10.3390/ijms26114990

**Published:** 2025-05-22

**Authors:** Evi Gkikopoulou, Christos-Chrysovalantis Syrigos, Ioanna Mantogiannakou, Chrysa-Eleni Petraki, Melina Stathopoulou, Melina Dragolia, Vagelis Rinotas, Vasileios Ntafis, Martina Rauner, Eleni Douni

**Affiliations:** 1 Institute for Bioinnovation, Biomedical Sciences Research Center “Alexander Fleming”, Fleming 34, 16672 Vari, Greece; gkikopoulou@fleming.gr (E.G.); syrigos@fleming.gr (C.-C.S.); stathopoulou@fleming.gr (M.S.);; 2 Laboratory of Genetics, Department of Biotechnology, Agricultural University of Athens, Iera Odos 75, 11855 Athens, Greece; 3 Institute for Fundamental Biomedical Research, Biomedical Sciences Research Center “Alexander Fleming”, Fleming 34, 16672 Vari, Greece; dragolia@fleming.gr (M.D.); ntafis@fleming.gr (V.N.); 4 Division of Endocrinology, Diabetes and Bone Diseases, Department of Medicine III and Center for Healthy Aging, University Medical Center, Technical University of Dresden, 01307 Dresden, Germany; martina.rauner@uniklinikum-dresden.de

**Keywords:** bone metastasis, breast cancer, RANKL, mouse models, pre-clinical models

## Abstract

Receptor activator of nuclear factor-κB ligand (RANKL) is essential for osteoclast formation and bone resorption, in osteolytic conditions such as osteoporosis and bone metastases. However, its role in metastasis progression remains incompletely understood. Herein, we examined whether the overexpression of human RANKL in transgenic mice (TgRANKL) affects their susceptibility to breast cancer bone metastasis compared to their wild-type (WT) littermates. Bone metastasis was induced by injecting EO771 mouse mammary adenocarcinoma cells into the caudal artery of syngeneic WT and TgRANKL mice. RANKL overexpression led to an earlier onset and increased burden of bone metastasis in EO771-bearing TgRANKL mice compared to WT mice. It also exacerbated the bone destruction caused by metastasis-associated osteolysis. The prophylactic inhibition of RANKL activity with denosumab, a monoclonal antibody targeting human RANKL, prevented osteolysis and significantly reduced the incidence and progression of bone metastases in TgRANKL mice. However, the therapeutic denosumab treatment had no effect on metastasis incidence or tumor burden, although it alleviated osteolysis. The treatment with zoledronic acid, an anti-resorptive agent inhibiting osteoclast activity, yielded results similar to those of denosumab. These findings emphasize the significance of initiating early treatment with anti-resorptive agents such as denosumab or zoledronic acid to reduce the risk of bone metastasis in patients at high risk.

## 1. Introduction

Breast cancer stands as one of the most prevalent forms of malignant tumors globally, posing the primary cause of mortality among women [1]. Despite advancements in early detection and clinical intervention, the mortality rate remains high due to treatment resistance and the spread of cancerous cells. Notably, approximately 70% of breast cancer patients, particularly in advanced cases, experience bone metastases [2]. The five-year overall survival rate for patients with bone metastases is critically low, at approximately 23% [3]. These patients frequently suffer from skeletal-related events (SREs), such as severe pain, pathological fractures, and hypercalcemia, all of which drastically impair their quality of life [4].

RANKL (receptor activator of nuclear factor-κB ligand), primarily produced by osteoblasts and osteocytes, is essential for the formation, activity, and survival of osteoclasts, the bone-resorbing cells [5,6]. RANKL binds to its receptor RANK on the surfaces of osteoclast precursors and activates downstream signaling cascades that promote their differentiation into mature osteoclasts. This process is crucial for the dynamic process of bone remodeling, ensuring bone integrity and calcium homeostasis [7]. Osteoprotegerin (OPG) acts as a decoy receptor for RANKL, inhibiting its interaction with RANK and thereby preventing the subsequent signaling events [8]. Apart from normal bone remodeling, RANKL also drives disease-related bone resorption, particularly in osteoporosis, where an imbalance in the RANKL-to-OPG ratio leads to excessive osteoclast activity and bone resorption, resulting in reduced bone strength and an increased risk of fractures [9]. In 2010, denosumab, a fully human IgG2 monoclonal antibody that specifically targets human RANKL, was approved for the treatment of postmenopausal osteoporosis in women at high fracture risk [10], as well as for the prevention of SREs, including fractures and spinal cord compression, in patients with bone metastases from solid tumors [11]. Denosumab remains a cornerstone in antiresorptive therapy, complementing other treatments such as bisphosphonates (BPs), which target and suppress osteoclast activity [12]. Zoledronic acid, a potent inhibitor among the newer generation of nitrogen-containing BPs, has a strong binding affinity to hydroxyapatite and promotes osteoclast apoptosis, effectively reducing the bone resorption and bone loss observed in osteoporotic patients [13].

The RANKL pathway has garnered renewed attention in recent years for its pivotal role in breast cancer progression and the development of bone metastases [14]. RANKL signaling promotes the proliferation of mammary epithelial and mammary stem cells, as well as the migration of breast cancer cells [15,16], while it also enhances osteoclast activity and bone resorption in bone metastasis. Breast-cancer-driven bone metastases primarily manifest as osteolytic lesions [17], characterized by increased bone resorption. This occurs due to a vicious cycle of interactions between breast cancer cells and the bone microenvironment. Mechanistically, among other factors, parathyroid-hormone-related peptide (PTHrP) released from bone metastatic cancer cells triggers RANKL expression in osteoblasts, which then promotes osteoclast formation and increased osteolysis [18]. The subsequent demineralization of the extracellular matrix results in the release of bone-stored growth factors, including transforming growth factor (TGF)-b, insulin-like growth factors (IGFs), and bone morphogenetic proteins (BMPs), which promote further cancer cell proliferation and create survival niches, establishing a vicious cycle of osteolytic metastasis [19]. While the role of RANKL in promoting osteoclast activation and bone resorption during bone metastasis is well-characterized, its effect in metastasis progression remains incompletely understood. Additionally, the clinical trial data investigating the role of anti-RANKL therapies in breast cancer and bone metastasis, beyond their impact on SREs, remain inconclusive [20,21]. This is mainly due to the fact that RANKL was primarily studied for its role in osteoclast-mediated bone resorption after metastasis establishment. In addition, the role of host-derived systemic RANKL as opposed to tumor-produced RANKL in metastatic dissemination, along with the ideal therapeutic window for intervention, remains poorly characterized.

We previously generated transgenic mice expressing human RANKL (huRANKL) using a 200 kb genomic fragment carrying the coding region and regulatory elements of the human *RANKL* gene, resulting in a physiologically relevant overexpression pattern that closely mirrors the expression profile of the endogenous mouse *Rankl* gene [22]. In TgRANKL mice, the overexpression of huRANKL leads to increased bone turnover, trabecular bone loss, cortical porosity, and the aberrant formation of bone marrow adiposity [23]. The TgRANKL mouse model serves as a novel preclinical tool to study how sustained host-derived RANKL production and pre-existing bone microenvironment changes influence metastatic risk. We hypothesize that elevated RANKL remodels the bone microenvironment and accelerates bone turnover, thereby promoting metastatic susceptibility, while early RANKL inhibition may prevent metastatic niche formation. Utilizing a transgenic mouse model that overexpresses huRANKL, we can directly evaluate the efficacy of denosumab, a monoclonal antibody specific to huRANKL and inactive in wild-type mice, thereby providing a clinically relevant platform for the assessment of human-targeted therapies. Unlike prior models that focus on RANKL inhibition in wild-type mice [5,24], our TgRANKL mouse model uniquely enables the direct testing of denosumab in mice.

In the present study, we investigated whether huRANKL overexpression affects the onset and progression of mammary-cancer-induced bone metastasis through the transfer of EO771 mouse mammary adenocarcinoma cells to syngeneic TgRANKL mice [25]. By transferring EO771 cells through the caudal artery, our model mimicked the hematogenous spread to bone, isolating RANKL’s effects on metastasis from primary tumor growth. We also evaluated whether prophylactic versus therapeutic RANKL inhibition influences metastatic progression, and whether osteoclast inhibition with zoledronic acid produces comparable effects, further elucidating the role of osteoclasts as key mediators. Our results demonstrate that RANKL overexpression enhanced mammary-cancer-driven bone metastasis, while its inhibition by denosumab significantly reduced bone metastases, showing comparable efficacy to zoledronic acid.

## 2. Results

### 2.1. TgRANKL Mice Develop Earlier Onset and Increased Burden of Mammary-Cancer-Induced Bone Metastasis

**Figure 1:**
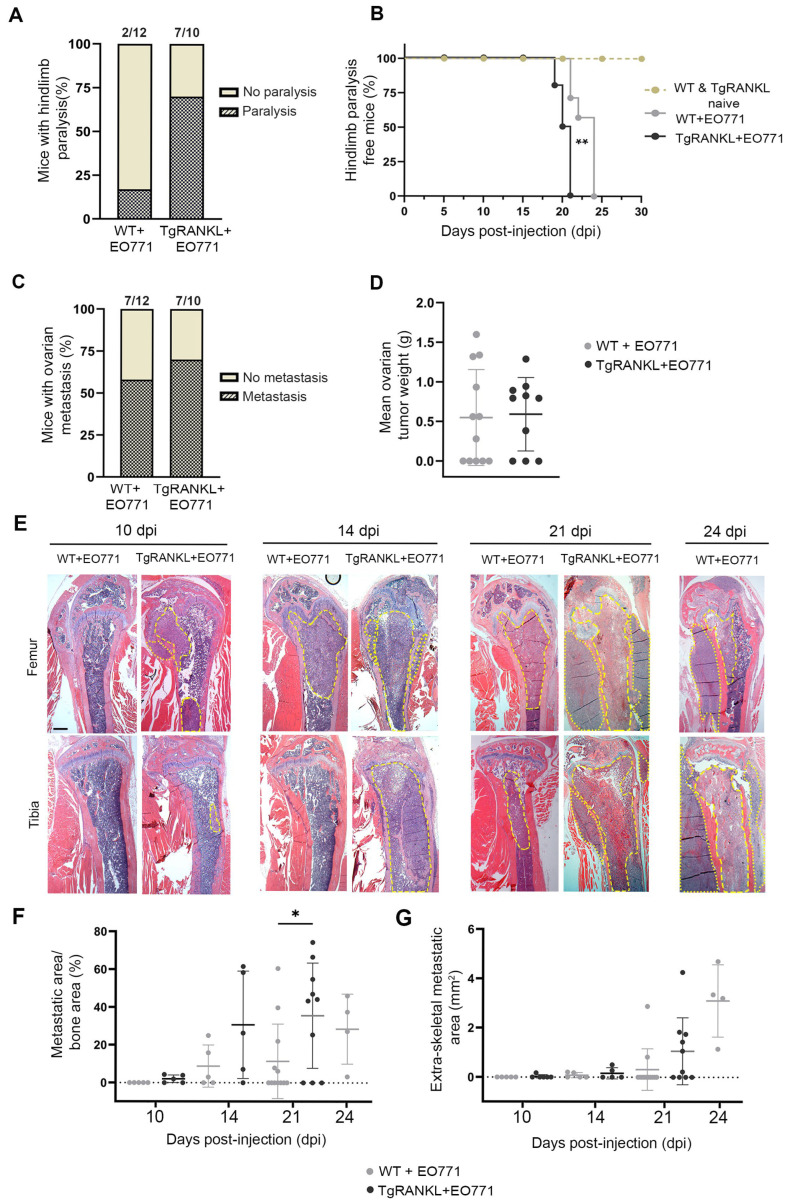
Earlier onset of mammary-cancer-driven bone metastasis and increased metastatic burden in TgRANKL mice. (**A**) Percentage and absolute number of WT+EO771 and TgRANKL+EO771 mice with metastasis-driven hindlimb paralysis at 21 dpi (n = 10–12/group). (**B**) Onset kinetics of hindlimb paralysis in mice (n = 6–10/group). Log-rank (Mantel-Cox) test was performed for the statistical analysis. (**C**) Percentage and absolute number of mice with post-mortem-confirmed ovarian metastasis and (**D**) mean ovarian tumor weight per mouse. Data are shown as mean values ± SD. Student’s *t*-test was performed for the statistical analysis (n = 10–12/group). (**E**) Representative histological images of femurs and tibiae stained with hematoxylin and eosin at the indicated dpi. Dashed yellow lines indicate the metastatic area within the bone, while dotted lines indicate the extraskeletal metastatic area. Scale bar, 150 μm. Quantification of (**F**) metastatic area/bone area and (**G**) extraskeletal metastatic area based in histological images of whole hindlimbs from WT+EO771 and TgRANKL+EO771 mice at the indicated dpi. Data are shown as mean values ± SD. Student’s *t*-test was performed for the statistical analysis between WT and TgRANKL mice at various dpi (n = 5–12/group), * *p* < 0.05, ** *p* < 0.001.

In the current study, we investigated whether huRANKL overexpression in TgRANKL mice affects the onset and progression of mammary-cancer-induced bone metastasis using EO771-Luc cells, a mouse mammary adenocarcinoma cell line with low metastatic potential [26] syngeneic to C57BL/6 mice, which stably expresses luciferase [27]. Bone metastasis was induced by injecting 1.4 × 10^5^ EO771-Luc cells through the caudal tail artery [28] in 8–10-week-old WT (WT+EO771) and TgRANKL (TgRANKL+EO771) female mice. Notably, by 21 dpi, 70% of the TgRANKL+EO771 mice developed hindlimb paralysis due to metastasis, compared to only 17% of the WT+EO771 mice (Figure 1A). In contrast, the majority of the WT+EO771 mice developed paralysis at a later stage (24 dpi) (Figure 1B), indicating a higher incidence of bone metastasis in the TgRANKL+EO771 group. Nevertheless, the metastasis of EO771 cells to the ovaries showed similar incidence and tumor size rates between the WT and TgRANKL mice (Figure 1C,D).

The histological analysis revealed metastatic regions in the femurs or tibiae of TgRANKL+EO771 mice as early as 10 dpi, while no metastatic foci were detected in the WT+EO771 group at this time point (Figure 1E,F). A higher metastatic burden was also noticed in the TgRANKL+EO771 mice compared to the WT+EO771 mice at 14 dpi reached statistical significance at 21 dpi within bone tissue (Figure 1E–G). In total, 70% of the TgRANKL+ΕO771 mice developed hindlimb metastasis compared to 42% of the WT+ΕO771 mice by 21 dpi (Figure 1E,F), highlighting the increased severity of metastatic progression in TgRANKL mice. Altogether, these findings indicate that huRANKL overexpression in transgenic mice expedites the progression of mammary-cancer-induced bone metastasis.

### 2.2. Severe Metastasis-Induced Osteolysis in TgRANKL Mice

**Figure 2:**
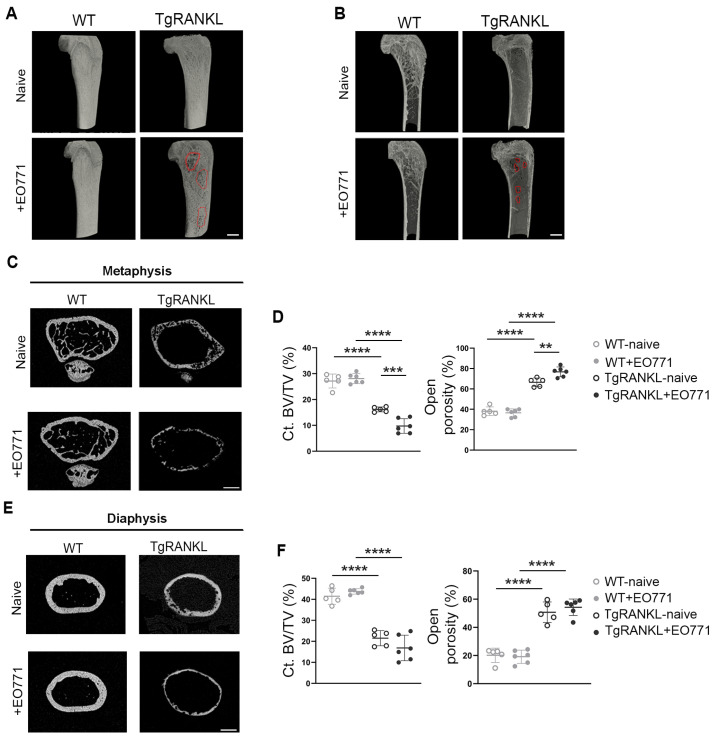
Enhanced mammary-cancer-induced osteolysis in TgRANKL mice. (**A**,**B**) Representative micro-CT 3D reconstructed longitudinal images of distal femurs from naïve and EO771-injected WT and TgRANKL mice at 21 dpi. Red lines highlight osteolytic areas. Scale bar, 1 mm. Representative 2D reconstructed cross-section images of the cortical bone at metaphysis (**C**) and diaphysis (**E**) with quantification (**D**–**F**) of cortical bone volume fraction (Ct.BV/TV, %) and total open porosity (%) values in femurs from each study group (n = 5–6/group). Scale bar, 500 μm. A two-way analysis of variance and Tukey’s post hoc test were performed for the statistical analysis; ** *p* < 0.01, *** *p* < 0.001, **** *p* < 0.0001.

To investigate the microarchitecture of metastatic bones, we conducted micro-CT scans on the distal femurs of EO771-injected WT and TgRANKL female mice at 21 dpi, while naïve mice from both genotypes served as controls. Three-dimensional (3D) reconstructed images of femurs from naïve mice confirmed the osteoporotic phenotype in the TgRANKL mice (Figure 2A,B), indicated by an almost complete absence of the trabecular network in the metaphyseal region (Figure 2C), along with cortical bone loss and increased open porosity both at metaphysis and diaphysis (Figure 2C–F). The bone microarchitecture remained largely unaffected in the WT+EO771 group. However, in the TgRANKL+EO771 mice, the bone metastasis caused extensive osteolysis, further aggravating their osteoporotic phenotype, as evidenced by the decreased Ct.BV/TV in the cortical bone and increased open porosity (Figure 2A–F, Table S1). Therefore, EO771-induced bone metastasis leads to severe bone destruction, characterized by large osteolytic areas, cortical bone loss, and open porosity in TgRANKL mice.

### 2.3. Prophylactic Anti-Resorptive Treatments Prevent Bone Metastasis and Osteolysis in TgRANKL Mice

**Figure 3:**
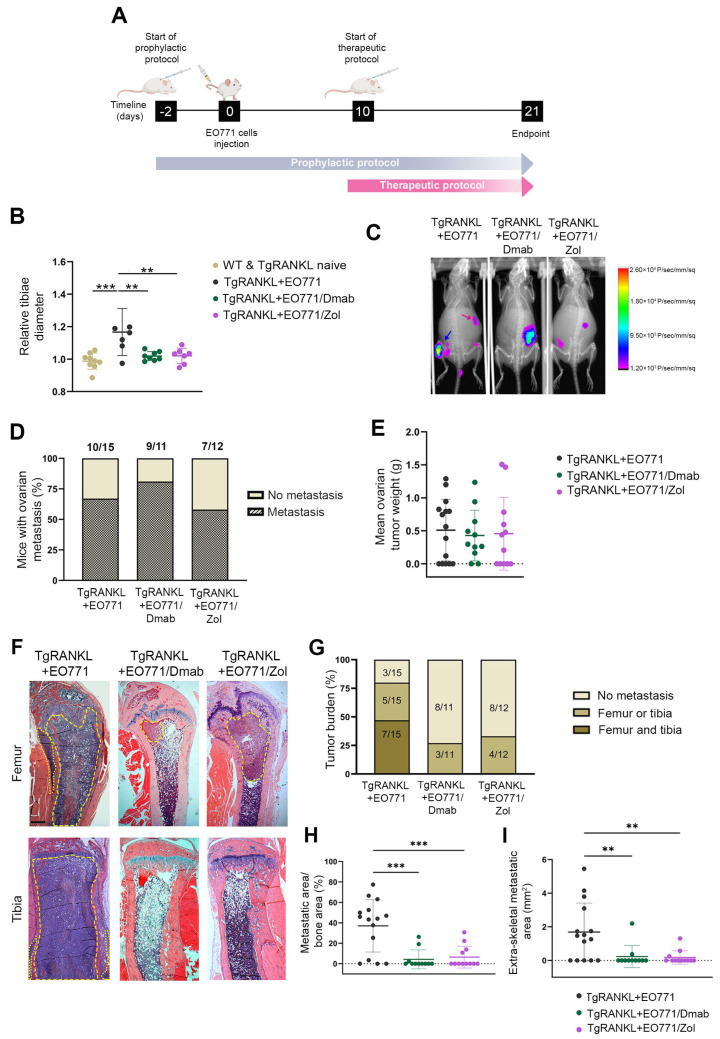
Prophylactic administration of denosumab or zoledronic acid prevents mammary-cancer-induced bone metastasis in TgRANKL mice. (**A**) Schematic representation of the EO771 bone metastasis model with denosumab (Dmab) and zoledronic acid (Zol) treatment strategies. All mice were euthanized at 21 dpi of cancer cells. (**B**) Relative diameter of the tibiae measured by caliper at the endpoint (n = 6–9/group). (**C**) Representative photos of in vivo bioluminescence imaging of TgRANKL+EO771, TgRANKL+EO771/Dmab, and TgRANKL+EO771/Zol mice at 19 dpi. Blue arrow indicates bone metastatic signal, while pink arrow indicates ovarian metastasis. (**D**) Percentage and absolute number of mice with post-mortem-confirmed ovarian metastasis and (**E**) mean ovarian tumor weight/mouse. (**F**) Representative images of femurs and tibiae stained with hematoxylin and eosin at 21 dpi. Dashed yellow lines indicate the metastatic area within the bone, while dotted lines indicate the extraskeletal metastatic areas. Scale bar, 150 μm. (**G**) Percentage and absolute number of mice with histologically verified metastatic tumors in femurs and tibiae. (**H**) Metastatic area/bone area in hindlimbs and (**I**) extraskeletal metastatic area (n = 11–15/group). Data are shown as mean values ± SD. A one-way ANOVA and Dunnett’s post hoc test were performed for the statistical analysis, ** *p* < 0.01, *** *p* < 0.001.

To demonstrate the role of huRANKL overexpression in promoting bone metastasis, we treated TgRANKL mice with denosumab, an anti-huRANKL monoclonal antibody [29]. In addition, by treating TgRANKL mice with zoledronic acid, which inhibits osteoclast proliferation and induces apoptosis [13], we investigated whether the direct blockade of osteolysis can impact the progression of bone metastasis. The study was designed to include a prophylactic treatment regimen, in which TgRANKL mice were administered subcutaneous injections of either 10 mg/kg of denosumab (TgRANKL+EO771/Dmab) or 0.5 mg/kg of zoledronic acid (TgRANKL+EO771/Zol) twice weekly, starting two days before EO771 cell injections and continuing for 21 days (Figure 3A).

Both the denosumab and zoledronic acid treatments prevented bone metastasis, as shown by a significant reduction in the tibiae diameter of the treated TgRANKL mice at 21 dpi (Figure 3B), which was further confirmed by in vivo bioluminescence imaging (Figure 3C). This highlights the effectiveness of anti-osteoporotic treatments in reducing the metastatic spread. However, the above treatments did not prevent ovarian metastasis in the TgRANKL mice (Figure 3D,E), suggesting that the mechanisms driving metastasis to the ovaries might be independent of RANKL.

A histological analysis of the femurs and tibiae at 21 dpi of cancer cells confirmed that both denosumab and zoledronic acid inhibited bone metastasis in TgRANKL mice (Figure 3F–H). Specifically, while 80% of the control TgRANKL+EO771 littermates developed bone metastases, the treatments with denosumab and zoledronic acid reduced the incidence rates to 27% and 33%, respectively (Figure 3G). Furthermore, the prophylactic treatment with denosumab and zoledronic acid effectively protected the TgRANKL mice from an increased metastatic burden, as indicated by the absence of metastases in both the femurs and tibiae of the treated groups, whereas 47% of the control mice developed metastases in both sites (Figure 3F,G). This is further supported by the fact that the denosumab and zoledronic acid treatments significantly decreased the metastatic burden not only in the hindlimbs (Figure 3H) but also in the surrounding soft tissue (Figure 3I).

**Figure 4:**
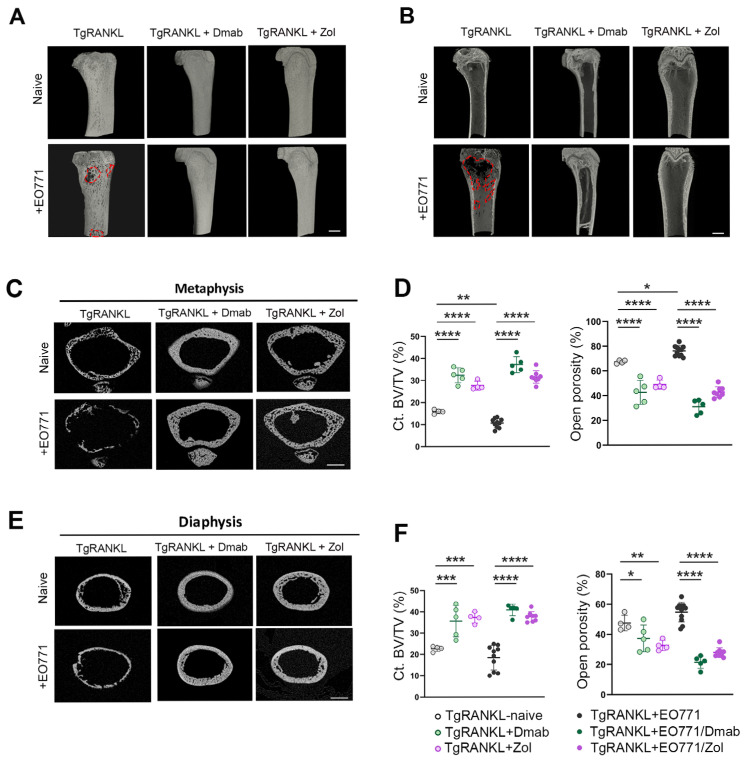
Prophylactic administration of denosumab or zoledronic acid attenuates osteolysis. (**A**,**B**) Representative micro-CT 3D reconstructed longitudinal images of distal femurs from naïve and EO771-injected TgRANKL mice, with or without prophylactic treatment with denosumab (Dmab) or zoledronic acid (Zol). Red lines highlight osteolytic areas. Scale bar, 1 mm. Representative 2D reconstructed cross-section images of the cortical bone at metaphysis (**C**) and diaphysis (**E**) with quantification (**D**–**F**) of the cortical bone volume fraction (Ct.BV/TV, %) and total open porosity (%) in femurs from each group (n = 4–10/group). Scale bar, 500 μm. Data are shown as mean values ± SD. A two-way ANOVA and Dunnett’s post hoc test were performed for the statistical analysis of more than two groups; * *p* < 0.05, ** *p* < 0.01, *** *p* < 0.001, **** *p* < 0.0001.

To examine the possible effect of the prophylactic treatments in metastasis-induced osteolysis, we analyzed the bones of EO771-injected mice with micro-CT at 21 dpi. The 3D reconstructed images showed that the denosumab and zoledronic acid treatments effectively protected the TgRANKL mice from huRANKL-mediated osteoporosis, as well as from metastasis-induced osteolysis (Figure 4). This finding was further supported by a significant increase in Ct.BV/TV and dramatically decreased open porosity in the metaphysis of the TgRANKL-treated mice (Figure 4C–F, Table S2). These results indicate that prophylactic treatments inhibiting either huRANKL or osteoclast activity protect the bones of TgRANKL+EO771 mice from osteolysis.

### 2.4. Effect of Therapeutic Anti-Resorptive Treatments in Bone Metastasis and Osteolysis

**Figure 5:**
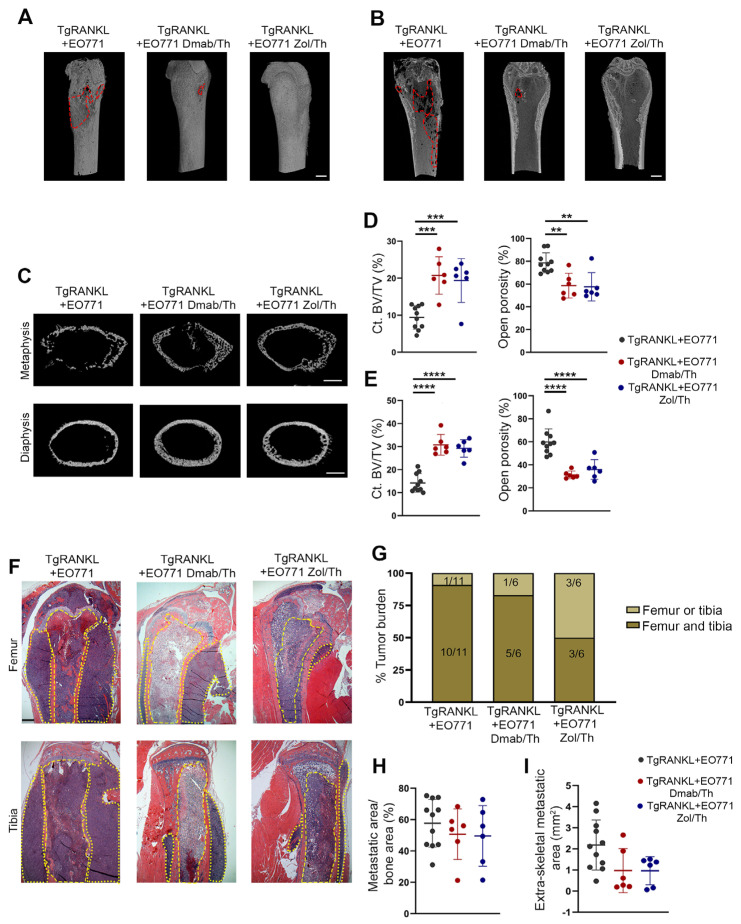
Therapeutic administration of denosumab or zoledronic acid prevents osteolysis but does not impact bone metastasis. (**A**,**B**) Representative micro-CT 3D reconstructed longitudinal images of distal femurs from naïve and EO771-injected TgRANKL mice, with or without therapeutic treatment with denosumab (Dmab) or zoledronic acid (Zol). Red lines highlight osteolytic areas. Scale bar, 1 mm. (**C**) Representative 2D reconstructed cross-section images of the cortical bone in the metaphysis with quantification (**D**,**E**) of the cortical bone volume fraction (Ct.BV/TV, %) and total open porosity (%) in femurs from each group (n = 6–10/group). Scale bar, 500 μm. (**F**) Representative images of femurs and tibiae stained with hematoxylin and eosin at 21 dpi. Dashed yellow lines indicate the metastatic area within the bone, while dotted lines indicate the extraskeletal metastatic areas. Scale bar, 150 μm. (**G**) Percentage and absolute number of each experimental group with histologically verified metastatic tumors in femurs and tibiae. (**H**) Metastatic area/bone area in hindlimbs and (**I**) extraskeletal metastatic area (n = 6–11/group). Data are shown as mean values ± SD. A one-way ANOVA and Dunnett’s post hoc test were performed for the statistical analysis of more than two groups; ** *p* < 0.01, *** *p* < 0.001, **** *p* < 0.0001.

We also examined whether therapeutic treatments of TgRANKL+EO771 mice with denosumab and zoledronic acid are effective against bone metastases. The treatments were initiated at 10 dpi of cancer cells, a time point where bone metastasis is already established in TgRANKL+EO771 mice (Figure 1E,F), and continued until 21 dpi. The 3D reconstructed micro-CT images of the cortical bones revealed that both treatments reduced osteolysis in the TgRANKL mice (Figure 5A,B). In this context, the denosumab and zoledronic acid therapies led to significantly increased Ct.BV/TV and reduced open porosity in the metaphysis and diaphysis of the TgRANKL+EO771 mice (Figure 5C–E, Table S3).

However, the histological analysis at 21 dpi revealed metastases in the hindlimbs across all experimental groups (Figure 5F,G), with comparable metastatic burden rates in the femurs and tibiae (Figure 5H). Nevertheless, treatment with denosumab or zoledronic acid led to a moderate reduction in tumor expansion within the surrounding tissue (Figure 5I). These findings suggest that the therapeutic treatments targeting huRANKL or osteoclast activity prevented SREs but did not impact established bone metastasis.

## 3. Discussion

In this study, we investigated whether the physiologically relevant upregulation of huRANKL expression in TgRANKL mice, beyond its role in osteolysis, influences the susceptibility to mammary-cancer-induced bone metastasis compared to their WT littermates. To induce bone metastasis, EO771 mammary cancer cells, which are characterized as a model for triple-negative breast cancer [27], were injected into the caudal tail artery of syngeneic mice. In this model, cancer cells predominantly colonize the bone marrow of the hindlimbs with much higher efficiency than via intracardiac injection, and with a shorter period of overt bone metastasis development [28]. Our findings demonstrate that RANKL overexpression leads to an earlier onset and increased burden of mammary-cancer-induced bone metastasis in EO771-bearing TgRANKL mice in comparison to WT mice. For the first time, our findings introduce an in vivo system that illustrates the effect of huRANKL overexpression on breast cancer bone metastasis.

The earlier onset of bone metastasis observed in the TgRANKL mice could be attributed to the chemoattractant properties of RANKL for breast cancer cells. Studies demonstrating that RANKL-producing osteoblasts, osteocytes, and bone marrow stromal cells [6,30] can attract and promote the migration of RANK-expressing tumor cells, such as EO771, offer a compelling explanation for the osteotropic nature of RANKL in breast cancer metastasis [5,24,31]. Additionally, it has been shown that RANKL enhances the expression of osteotropic genes in cancer cells, including matrix metalloproteinases (MMPs) and interleukins [24], thereby promoting osteoclastogenesis. In this context, it is worth noting that EO771 mammary cancer cell metastasis to non-bone tissues, such as the ovaries, remains unaffected by the overexpression of huRANKL, highlighting RANKL′s specificity in driving bone metastasis. In TgRANKL mice, apart from the bone tissue, huRANKL is also expressed by bone marrow adipocytes [23], which could further promote osteotropic metastasis.

In addition, the early colonization and tumor expansion observed in TgRANKL mice could be related to the RANKL-driven osteoporotic bone microenvironment, characterized by enhanced osteoclast activity and bone remodeling [22], creating a favorable environment for tumor cell growth within the bone [19,32]. The interplay between tumor cells and the bone microenvironment establishes a reciprocal “vicious cycle” of bone destruction, where increased osteoclastic activity results in bone destruction, which in turn releases growth factors that promote further tumor growth and metastasis [33]. This finding aligns with studies showing that ovariectomy-induced bone resorption enhances the growth of disseminated MDA-MB-231 breast cancer cells in BALB/c nude mice, which is prevented by RANKL inhibition with OPG-Fc treatment [34]. Similarly, patients with precancerous osteoporosis are more likely to present with disseminated tumor cells in their bone marrow than patients with normal or osteopenic bone density [35]. Untreated osteoporosis has also been linked to the accelerated progression of bone metastases, although precancer osteoporosis was not associated with the risk of bone metastasis [36].

Apart from an increased bone metastasis burden, the TgRANKL mice also developed extensive osteolysis, exacerbating their osteoporotic phenotype, which could be correlated with increased osteoclastogenesis. Increased RANKL levels may cause enhanced osteoclast activity and promote tumor cell invasion [14], consistent with previous studies implicating the RANKL–RANK axis in driving osteolysis during breast cancer metastasis [5,37,38]. This is mediated by increased stromal RANKL and osteoclastogenesis-inducing factors such as IL-6, IL-8, IL-11, MMP-1, and MMP-13 [24]. In support of this, Nannuru et al. demonstrated that the tumor–bone interface, a region with significant osteoclast activity, shows high RANKL expression, in contrast to the lower RANKL expression in the metastatic area itself [39]. Beyond preclinical models, the evidence suggests that human tumor cells can secrete factors into the bone microenvironment, including IL-6 and PTH-related peptide (PTHrP), which increase the RANKL-to-OPG ratio, thereby enhancing osteolysis and promoting tumor progression [40].

Denosumab, a human monoclonal antibody targeting RANKL, is clinically used to inhibit RANKL–RANK interactions. It is approved for the treatment of osteoporosis in postmenopausal women at high risk for fractures but also to prevent SREs such as fractures and bone pain in patients with multiple myeloma or bone metastases from solid tumors [10,41,42]. However, the findings of denosumab treatment in women with early-stage breast cancer as regards bone metastasis remain currently inconclusive, based on conflicting data from two large cohort, placebo-controlled studies with fundamental differences in patient characteristics, design, and denosumab dosing. In particular, the ABCSG-18 trial assessed the efficacy of denosumab alongside aromatase inhibitors in post-menopausal women with early breast cancer, showing an improvement in disease-free survival, including for bone metastases [20]. In contrast, the D-CARE trial assessed the effects of denosumab in a cohort of women starting standard neoadjuvant or adjuvant chemotherapy, regardless of their menopausal or hormone receptor status [21]. No difference in the composite primary end points of bone metastasis-free survival was demonstrated after at least 5 years of follow-up monitoring, while the bone-related outcomes were improved [43]. In the current study, we utilized a unique bone metastasis model with huRANKL overexpression, enabling a direct preclinical evaluation of denosumab, unlike previous models targeting endogenous mouse RANKL [5,24,37,38]. We showed that the prophylactic administration of denosumab not only inhibited osteolysis in TgRANKL mice but effectively diminished the occurrence and progression of bone metastasis, supporting the potential use of denosumab as a preventive treatment in breast cancer patients with high risk of bone metastasis. Our findings are consistent with preclinical data demonstrating the efficacy of RANKL blockade in reducing breast-cancer-induced bone metastasis [5,34,38,44]. One of the most significant novel findings is the clear demonstration of differential outcomes between prophylactic and therapeutic denosumab treatments. The therapeutic treatment of TgRANKL mice with denosumab did not reduce the metastasis incidence or tumor burden compared to the control mice, while it mitigated osteolysis and restored the cortical bone volume well beyond the levels observed in the osteoporotic TgRANKL mice, emphasizing its role in managing SREs, even in the context of advanced disease progression. Notably, neither huRANKL overexpression in TgRANKL mice nor denosumab treatment impacted the metastasis of EO771 cells to the ovaries, which is consistent with other reports indicating that the RANK–RANKL axis does not affect metastasis to organs other than the skeleton [38,45].

In addition to denosumab, other anti-resorptive drugs have been widely studied for managing bone metastases in breast cancer patients. Zoledronic acid, commonly used in clinical settings, was tested in TgRANKL mice using both prophylactic and therapeutic approaches, showing similar results as with denosumab, underscoring the importance of anti-resorptive therapies in the colonization and progression of bone metastasis. Zoledronic acid also seems to reduce both the skeletal and visceral tumor burden in mouse models of breast cancer through effects on tumor cell migration and invasion [46]. Zoledronic acid has been also studied for its clinical benefits in breast cancer patients with bone metastases. A placebo-controlled trial on Japanese women with breast-cancer-induced bone metastasis confirmed the significant clinical benefits of zoledronic acid by reducing the SRE ratio within a year of the treatment, as well as by delaying the time of the first SRE’s occurrence [47]. Moreover, the ABCSG-12 trial, which examined the benefits of zoledronic acid addition to the combinatory effect of goserelin and tamoxifen/anastrozole in premenopausal patients with early ER+ breast cancer, revealed that that zoledronic acid improved the disease-free survival mostly in patients aged 40 or older [48]. The AZURE trial, which enrolled more advanced breast cancer patients and investigated the possible effect of zoledronic acid addition in standard adjuvant therapy, demonstrated that zoledronic acid reduced bone metastasis and improved outcomes in patients with established menopause, although it did not provide an overall benefit. Denosumab has been shown to be more effective than zoledronic acid in delaying the time to the development of SREs in patients with bone metastases [49]. However, the clinical recommendations still support the use of adjuvant bisphosphonate therapy, particularly zoledronic acid, for postmenopausal patients with high-risk breast cancer to reduce the risk of distant metastasis [50]. Our study’s design, which evaluates two different treatments administered at distinct time points within the same experimental setting, enables the comparison of their efficacy and timing, thereby providing critical insights for optimizing therapeutic strategies against bone metastasis. This timing-dependent efficacy of denosumab and zoledronic acid represents a crucial clinical insight not previously demonstrated in experimental models of breast cancer bone metastasis. Therefore, additional clinical data are required to clarify the potential protective benefits of huRANKL inhibitors against bone metastasis.

## 4. Materials and Methods

### 4.1. Mouse Husbandry

TgRANKL (Tg5519 line) [22] and WT control female mice of a C57BL/6 background were maintained and bred in the animal facility of the Alexander Fleming Biomedical Sciences Research Center under specific pathogen-free conditions. All animal work was approved by the Institutional Protocol Evaluation Committee and was licensed by the Veterinary Authorities of Attica Prefecture in compliance with PD 56/2013 and European Directive 2010/63/EU.

### 4.2. EO771 Cell Line Culture

The mouse mammary adenocarcinoma cell line EO771-Luc that stably expresses luciferase was kindly provided by Dr. Martina Rauner (Technische Universität Dresden, Dresden, Germany) [27]. The cells were cultured in DMEM (Gibco Thermo Fisher Scientific, Waltham, MA, USA) supplemented with 10% fetal bovine serum (FBS) (Avantor, Radnor, PA, USA) and 1% penicillin/streptomycin (Gibco Thermo Fisher Scientific, Waltham, MA, USA). The cells were frozen in a solution of 10% dimethyl sulfoxide (DMSO) (Sigma-Aldrich, St. Louis, MO, USA) in FBS and stored at −80 °C.

### 4.3. Bone Metastasis Model

Here, 1.4 × 10^5^ EO771-Luc cells in 100 μL of phosphate-buffered saline (PBS) were injected into the caudal tail artery of 8–10-week-old female WT and TgRANKL mice [28]. The mice were monitored for metastasis progression via in vivo bioluminescence imaging at 19 dpi, as well as through histological analyses at various time points. Mice experiencing difficulty walking were monitored daily, and the timing of hindlimb paralysis was documented. Extraosseous metastasis for the TgRANKL mice was assessed at the endpoint using a digital caliper, with measurements taken just below the knee level.

### 4.4. Denosumab and Zoledronic Acid Treatments

In the prophylactic treatments, the TgRANKL mice were treated twice weekly subcutaneously with 10 mg/kg of denosumab (Amgen Inc., Thousand Oaks, CA, USA) or 0.5 mg/kg of zoledronic acid (Aclasta, Novartis Pharmaceuticals, Basel, Switzerland), starting 2 days prior to cancer cell injection until 21 days post-injection (dpi) of cancer cells. In the therapeutic regimen, the TgRANKL mice received subcutaneous injections twice weekly of either 30 mg/kg of denosumab or 0.5 mg/kg of zoledronic acid, starting 10 dpi of cancer cells, upon the establishment of bone metastasis, and continuing until 21 dpi. The selected doses for both agents were based on body surface area conversion [51,52] from clinically relevant human doses [20,21,47]. Specifically for denosumab, we selected a prophylactic dose of 10 mg/kg administered twice weekly, based on previous studies using our TgRANKL model [22,53]. For therapeutic administration, the dose was increased to 30 mg/kg to assess whether inhibition could be achieved in the context of established bone metastases. Regarding zoledronic acid, most studies use 0.1–0.5 mg/kg as clinically relevant doses in mice [54,55,56]. In our study, we selected the higher dose (0.5 mg/kg) for the TgRANKL osteoporotic model, which exhibits severe bone loss, to ensure the strong suppression of bone turnover and to evaluate the upper limit of the drug’s efficacy. The hindlimbs and ovaries were collected at the endpoint of the studies.

### 4.5. In Vivo Imaging

Metastasis was assessed at 19 dpi of cancer cells using in vivo bioluminescence imaging (In-Vivo Xtreme, Bruker, Ettlingen, Germany). Seventeen minutes prior to imaging, all mice were intraperitoneally injected with 150 mg/kg of D-luciferin (PerkinElmer, Inc., Shelton, CT, USA) in PBS. The imaging conditions were as follows: an exposure time of 2 min, binning at 8 × 8 pixels, a field of view of 19 cm, and an f/stop of 1.1.

### 4.6. Histological Examination

The bones were fixed in 10% neutral-buffered formalin (Carlo Erba Reagents, S.r.l., Cornaredo, Italy) overnight, decalcified in 13% EDTA, and embedded in paraffin. Sections measuring 4 μm in thickness were stained with hematoxylin and eosin. The histology images were acquired using a Nikon E800 upright widefield fluorescence microscope (Nikon Instruments Inc., Tokyo, Japan). The whole bone area of the femurs and tibiae and the metastatic area were measured with the open-source software ImageJ (version 1.53c). The metastatic burden was assessed as metastatic area/bone area (%).

### 4.7. Micro-CT Analysis

The femurs were fixed in 10% neutral-buffered formalin (Carlo Erba Reagents, S.r.l., Cornaredo, Italy) overnight, washed with water, and stored in PBS at 4 °C. The microarchitecture of the distal femurs from all experimental mice was evaluated through micro-CT (SkyScan1172, Bruker, Ettlingen, Germany). Images were acquired at 50 KV, 100 µA with a 0.5 mm aluminum filter and 6 μm voxel size. Three-dimensional reconstructions (8.8 mm cubic resolution) were generated using NRecon software (Version 1.7.4.2, Bruker), as previously described [53]. The femoral cortical geometry was assessed with CTAn software (Version 1.18.8.0+, Bruker) using 300 continuous CT slices (1800 µm) for the metaphyseal area underneath the growth plate and using 100 continuous CT slices (600 µm) for the area of the diaphysis. For each area, the cortical bone volume fraction (Ct. BV/TV, %) and percent open porosity were assessed. The percent open porosity reflects the volume fraction of the interconnected pores that extends to the external surface (such as canals and microcracks) relative to the total volume of cortical bone. The three-dimensional imaging was performed using CTVox software (version 3.3.0 r1403, Bruker).

### 4.8. Statistical Analysis

All results are presented as scatter dot-plots, showing each data point as the mean value ± standard deviation (SD). Statistical significance was assessed using a one-way analysis of variance (ANOVA) or two-way analysis of variance (ANOVA) with Tukey or Dunnett’s post hoc test to compare the means of multiple groups. Dunnett’s test was used to compare groups included in treatments. Moreover, Student’s *t*-test was used for two-group comparisons. A log-rank (Mantel-Cox) test was performed for the statistical analysis of the onset kinetics of metastasis-driven hindlimb paralysis. For all tests, *p* < 0.05 was considered statistically significant.

## 5. Conclusions

In conclusion, this study provides novel evidence establishing a causal link between RANKL overexpression and increased metastatic burden. It demonstrates the specific therapeutic effect of denosumab in TgRANKL mice, reveals a correlation between bone loss and metastatic progression, and distinguishes the differential efficacy of prophylactic versus therapeutic interventions. Furthermore, the comparative analysis of denosumab and zoledronic acid underscores the critical importance of the timely administration of anti-resorptive therapy in mitigating metastatic bone disease. Our results reinforce the therapeutic promise of targeting the RANKL pathway or osteoclast function as a preventive strategy in breast-cancer-induced bone metastasis. Such early interventions could not only preserve the skeletal integrity but also affect the progression of bone metastases in patients at increased risk of bone metastases.

## Data Availability

Data is contained within the article and Supplementary Materials.

## Supplementary Materials

The supporting information can be downloaded at: https://www.mdpi.com/article/10.3390/ijms26114990/s1.

## Abbreviations

The following abbreviations are used in this manuscript:

Ct.BV/TV	Cortical bone volume/total volume
Dmab	Denosumab
dpi	Days post-injection
OPG	Osteoprotegerin
RANK	Receptor activator of nuclear factor-κB
RANK	Receptor activator of nuclear factor-κB ligand
SREs	Skeletal-related events
WT	Wild-type
Zol	Zoledronic acid
