# Supplementary material for: RANKL Drives Bone Metastasis in Mammary Cancer: Protective Effects of Anti-Resorptive Treatments
Source: Int J Mol Sci. 2025 May 22;26(11):4990. doi: 10.3390/ijms26114990 (PMC12155363; doi:10.3390/ijms26114990)
Supplement: Supplementary file 1 [file ijms-26-04990-s001.zip › ijms-3635907-supplementary.pdf]

RANKL Drives Bone Metastasis in Mammary Cancer: Protective Effects of Anti-Resorptive Treatments

Evi Gkikopoulou [1,2], Christos-Chrysovalantis Syrigos [1,2], Ioanna Mantogiannakou [1], Chrysa-Eleni Petraki [1,2], Melina Stathopoulou [1,2], Melina Dragolia [3], Vagelis Rinotas [1], Vasileios Ntafis [3], Martina Rauner [4] and Eleni Douni [1,2,*]

[1] Institute for Bioinnovation, Biomedical Sciences Research Center "Alexander Fleming", Fleming 34, 16672 Vari, Greece
[2] Laboratory of Genetics, Department of Biotechnology, Agricultural University of Athens, Iera Odos 75, 11855 Athens, Greece
[3] Institute for Fundamental Biomedical Research, Biomedical Sciences Research Center "Alexander Fleming", Fleming 34, 16672 Vari, Greece
[4] Division of Endocrinology, Diabetes and Bone Diseases, Department of Medicine III and Center for Healthy Aging, University Medical Center, Technical University of Dresden, 01307 Dresden, Germany
* Correspondence: douni@aua.gr

Table S1. Femoral bone parameters through micro-CT analysis in naïve (WT and TgRANKL) mice, WT+EO771 and TgRANKL+EO771 mice (n=5-6/group). Two-way analysis of variance (ANOVA) and Tuckey's post-hoc test was performed for statistical analysis. Data are presented as mean values ± SD. [a] *p*<0.05 vs WT-naive, [b] *p*<0.05 vs TgRANKL-naive, [c] *p*<0.05 vs WT+EO771. Cortical bone structure was assessed with Ct.BV/TV, cortical bone volume/tissue volume; Ct.BV, cortical bone volume; Open Porosity, open cortical porosity in metaphysis and diaphysis.

**Femoral areas**	**Femoral Parameters**	**WT-naive**	**WT+EO771**	**TgRANKL-naive**	**TgRANKL+ EO771**
**Metaphysis**	Ct.BV/TV (%)	27.15 ± 2.7	28.08 ± 1.937	16.17 ± 0.96 [a]	9.77 ± 2.82 [a,b,c]
	Open Porosity (%)	38.15 ± 4.24	36.56 ± 53.84	66.48 ± 4.27 [a]	76.69 ± 4.82 [a,b,c]
**Diaphysis**	Ct.BV/TV (%)	41.49 ± 3.84	43.76 ± 1.30	21.52 ± 3.63 [a]	16.84 ± 6.08 [a,c]
	Open Porosity (%)	20.13 ± 5.13	19.16 ± 4.7	50.72 ± 7.28 [a]	54.26 ± 5.84 [a,c]

Table S2. Femoral bone parameters through micro-CT analysis in TgRANKL naïve and TgRANKL+EO771 mice either treated or not with denosumab (Dmab) and zoledronic acid (Zol) prophylactically (n=4-10/group). Two-way analysis of variance (ANOVA) and Dunnett post-hoc test was performed for statistical analysis. Data are presented as mean values ± SD. $^{a}p<0.05$ vs TgRANKL-naive, $^{b}p<0.05$ vs TgRANKL+EO771. Cortical bone structure was assessed with Ct.BV/TV, cortical bone volume/tissue volume; Ct.BV, cortical bone volume; Open Porosity, open cortical porosity in metaphysis and diaphysis.

**Femoral area**	**Parameters**	**TgRANKL-naive**	**TgRANKL +Dmab**	**TgRANKL + Zol**	**Tg RANKL +EO771**	**TgRANKL +EO771/ Dmab**	**TgRANKL +EO771/ Zol**
**Metaphysis**	Ct.BV/TV (%)	15.87 ± 0.95 [b]	32.35 ± 3.40 [a,b]	27.65 ± 1.99 [a,b]	10.58 ± 2.08 [a]	37.17 ± 3.64 [b]	31.47 ± 2.83 [a,b]
	Open Porosity (%)	67.4 ± 1.23 [b]	42.60 ± 9.72 [a,b]	48.89 ± 3.71 [a,b]	76.11 ± 4.08[a]	30.94 ± 5.68 [b]	42.79 ± 4.35 [a,b]
**Diaphysis**	Ct.BV/TV (%)	22.68 ± 1.28	35.63 ± 7.40 [a,b]	37.26 ± 2.59 [a,b]	18.45 ± 5.79	40.82 ± 2.66 [b]	37.63 ± 2.39 [a,b]
	Open Porosity (%)	47.51 ± 5.22	37.27 ± 8.91 [a,b]	32.53 ± 3.44 [a,b]	54.69 ± 6.45	21.44 ± 4.13 [b]	27.82 ± 3.31 [a,b]

Table S3. Femoral bone parameters through micro-CT analysis in TgRANKL+EO771 mice treated with denosumab (Dmab) and zoledronic acid (Zol) therapeutically (n=6-10/group). One-way analysis of variance (ANOVA) and Dunnett post-hoc test was performed for statistical analysis. Data are presented as mean values ± SD. [a]*p*<0.05 vs TgRANKL+EO771. Cortical bone structure was assessed with Ct.BV/TV, cortical bone volume/tissue volume; Ct.BV, cortical bone volume; Open Porosity, open cortical porosity in metaphysis and diaphysis.

**Femoral area**	**Parameters**	**TgRANKL + EO771**	**TgRANKL + EO771/ Dmab-Th**	**TgRANKL + EO771/ Zol-Th**
**Metaphysis**	Ct.BV/TV (%)	9.42 ± 3.17	20.73 ± 5.07[a]	19.38 ± 5.92[a]
	Open Porosity (%)	78.66 ± 8.80	58.61 ± 10.84[a]	57.62 ± 12.40[a]
**Diaphysis**	Ct.BV/TV (%)	14.23 ± 4.05	30.78 ± 4.51[a]	29.22 ± 3.82[a]
	Open Porosity (%)	59.90 ± 11.41	31.31 ± 3.34[a]	35.93 ± 8.58[a]
